# Bis(diisopropyl­ammonium) naphthalene-1,5-disulfonate

**DOI:** 10.1107/S1600536811043492

**Published:** 2011-10-29

**Authors:** Yu Jin

**Affiliations:** a Ordered Matter Science Research Center, Southeast University, Nanjing 211189, People’s Republic of China

## Abstract

In the title compound, 2C_6_H_16_N^+^·C_10_H_6_O_6_S_2_^2−^, the cations and anions are associated *via* N—H⋯O and C—H⋯O hydrogen-bonding inter­actions.

## Related literature

For general background on ferroelectric metal–organic frameworks, see: Fu *et al.* (2009 ▶); Wu *et al.* (2011 ▶); Ye *et al.* (2006 ▶); Zhang *et al.* (2008 ▶, 2010 ▶).
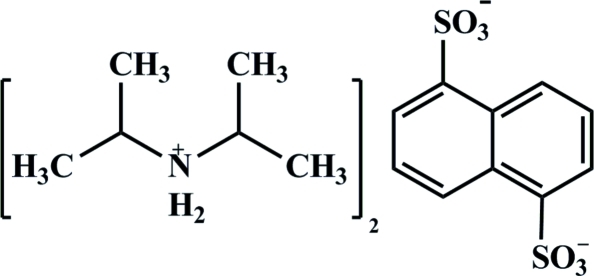


## Experimental

### 

#### Crystal data

2C_6_H_16_N^+^·C_10_H_6_O_6_S_2_^2−^
*M* *_r_* = 490.66
Triclinic, 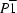

*a* = 7.9518 (16) Å
*b* = 9.1215 (18) Å
*c* = 9.4319 (19) Å
α = 74.33 (3)°
β = 88.60 (3)°
γ = 74.74 (3)°
*V* = 634.7 (2) Å^3^
*Z* = 1
Mo *K*α radiation
μ = 0.25 mm^−1^
*T* = 293 K
0.3 × 0.3 × 0.2 mm

#### Data collection

Rigaku Mercury CCD diffractometer
Absorption correction: multi-scan (*CrystalClear*; Rigaku, 2005 ▶) *T*_min_ = 0.489, *T*_max_ = 1.000
6562 measured reflections
2904 independent reflections
2621 reflections with *I* > 2σ(*I*)
*R*_int_ = 0.038

#### Refinement

*R*[*F* ^2^ > 2σ(*F* ^2^)] = 0.046
*wR*(*F* ^2^) = 0.129
*S* = 1.12
2904 reflections
150 parameters
H-atom parameters constrained
Δρ_max_ = 0.50 e Å^−3^
Δρ_min_ = −0.45 e Å^−3^

### 

Data collection: *CrystalClear* (Rigaku, 2005 ▶); cell refinement: *CrystalClear*; data reduction: *CrystalClear*; program(s) used to solve structure: *SHELXS97* (Sheldrick, 2008 ▶); program(s) used to refine structure: *SHELXL97* (Sheldrick, 2008 ▶); molecular graphics: *SHELXTL* (Sheldrick, 2008 ▶); software used to prepare material for publication: *SHELXL97*.

## Supplementary Material

Crystal structure: contains datablock(s) I, global. DOI: 10.1107/S1600536811043492/mw2025sup1.cif

Structure factors: contains datablock(s) I. DOI: 10.1107/S1600536811043492/mw2025Isup2.hkl

Supplementary material file. DOI: 10.1107/S1600536811043492/mw2025Isup3.cml

Additional supplementary materials:  crystallographic information; 3D view; checkCIF report

## Figures and Tables

**Table 1:** Hydrogen-bond geometry (Å, °)

*D*—H⋯*A*	*D*—H	H⋯*A*	*D*⋯*A*	*D*—H⋯*A*
N1—H1*C*⋯O1^i^	0.90	2.03	2.887 (2)	159
N1—H1*D*⋯O3^ii^	0.90	2.02	2.916 (2)	174
C9—H9*A*⋯O3	0.96	2.58	3.480 (3)	156
C6—H6*A*⋯O3^ii^	0.96	2.58	3.351 (3)	138
C11—H11*C*⋯O1^ii^	0.96	2.61	3.439 (4)	144
C11—H11*B*⋯O2^i^	0.96	2.47	3.382 (3)	158

Symmetry codes: (i) 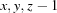; (ii) 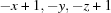.

**Fig. 1.**
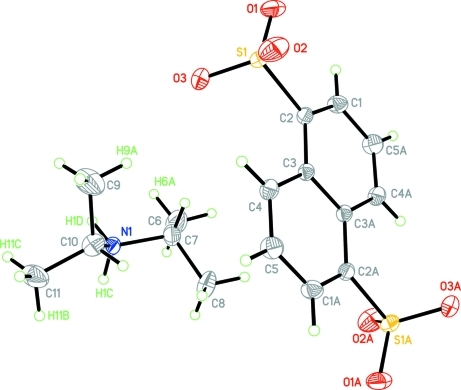
Perspective view of one cation and the anion for (I).Displacement ellipsoids drawn at the 30% probability level.

**Fig. 2.**
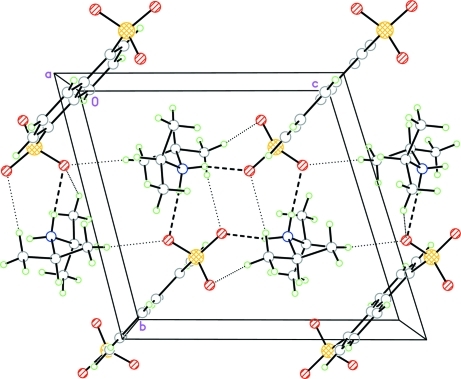
Packing of (I) viewed down the a axis. The N—H···O and C—H···O interactions are shown, respectively as dashed and dotted lines.

## supplementary crystallographic information

### Comment

Currently, simple molecular-ionic compounds containing organic cations and anions are of considerable interest owing to the tunability of their structural features and their potential to show ferroelectric properties. There exists a series of compounds in which the components can be arranged in a disordered fashion at a relative high temperature and in an ordered fashion at a relative low temperature. (Fu *et al.*, 2009; Zhang *et al.*, 2010; Zhang *et al.*, 2008;Ye *et al.*, 2006). The transition from the disordered arrangement to the ordered one leads to sharp change in the physical properties of the compound. As part of our search for simple ferroelectric compounds we have investigated the title compound and report its room temperature structure.

The centrosymmetric anion and one cation are shown in Fig. 1 with the hydrogen bonds listed in Table 1. These interactions tie the cations and anions together in sheets approximately parallel to {100} with zig-zag rows of cations lying between rows of anions (Fig. 2). There are only van der Waals interactions between layers.

### Experimental

1,5-naphthalenedisulfonic acid (10 mmol, 2.88 g) was dissolved in 15 ml of distilled water and was stirred for 5 minutes after which diisopropylamine (1.5 ml) was added with stirring. The solution was filtered and left to stand undisturbed whereupon colorless block crystals suitable for X-ray diffraction were obtained in about 68% yield after two days. These were filtered off and washed with distilled water.

### Refinement

H atoms bound to carbon and nitrogen were placed at idealized positions [C—H = 0.93–0.96 Å and N—H = 0.90 Å] and allowed to ride on their parent atoms with *U*_iso_ fixed at 1.2 *U*_eq_(C,*N*).

### Crystal data

**Table:**

2C_6_H_16_N^+^·C_10_H_6_O_6_S_2_^2^^−^	*Z* = 1
*M**_r_* = 490.66	*F*(000) = 264
Triclinic, *P*1	*D*_x_ = 1.284 Mg m^−^^3^
Hall symbol: -P 1	Mo *K*α radiation, λ = 0.71073 Å
*a* = 7.9518 (16) Å	Cell parameters from 3450 reflections
*b* = 9.1215 (18) Å	θ = 3.1–27.6°
*c* = 9.4319 (19) Å	µ = 0.25 mm^−^^1^
α = 74.33 (3)°	*T* = 293 K
β = 88.60 (3)°	Block, colorless
γ = 74.74 (3)°	0.3 × 0.3 × 0.2 mm
*V* = 634.7 (2) Å^3^	

### Data collection

**Table:**

Rigaku Mercury CCD diffractometer	2904 independent reflections
Radiation source: fine-focus sealed tube	2621 reflections with *I* > 2σ(*I*)
graphite	*R*_int_ = 0.038
ω scans	θ_max_ = 27.5°, θ_min_ = 3.1°
Absorption correction: multi-scan (*CrystalClear*; Rigaku, 2005)	*h* = −10→10
*T*_min_ = 0.489, *T*_max_ = 1.000	*k* = −11→11
6562 measured reflections	*l* = −12→12

### Refinement

**Table:**

Refinement on *F*^2^	Secondary atom site location: difference Fourier map
Least-squares matrix: full	Hydrogen site location: inferred from neighbouring sites
*R*[*F*^2^ > 2σ(*F*^2^)] = 0.046	H-atom parameters constrained
*wR*(*F*^2^) = 0.129	*w* = 1/[σ^2^(*F*_o_^2^) + (0.0558*P*)^2^ + 0.2055*P*] where *P* = (*F*_o_^2^ + 2*F*_c_^2^)/3
*S* = 1.12	(Δ/σ)_max_ < 0.001
2904 reflections	Δρ_max_ = 0.50 e Å^−^^3^
150 parameters	Δρ_min_ = −0.45 e Å^−^^3^
0 restraints	Extinction correction: *SHELXL97* (Sheldrick, 2008), Fc^*^=kFc[1+0.001xFc^2^λ^3^/sin(2θ)]^-1/4^
Primary atom site location: structure-invariant direct methods	Extinction coefficient: 0.85 (3)

### Special details

**Table:**

Geometry. All e.s.d.'s (except the e.s.d. in the dihedral angle between two l.s. planes) are estimated using the full covariance matrix. The cell e.s.d.'s are taken into account individually in the estimation of e.s.d.'s in distances, angles and torsion angles; correlations between e.s.d.'s in cell parameters are only used when they are defined by crystal symmetry. An approximate (isotropic) treatment of cell e.s.d.'s is used for estimating e.s.d.'s involving l.s. planes.
Refinement. Refinement of *F*^2^ against ALL reflections. The weighted *R*-factor *wR* and goodness of fit *S* are based on *F*^2^, conventional *R*-factors *R* are based on *F*, with *F* set to zero for negative *F*^2^. The threshold expression of *F*^2^ > σ(*F*^2^) is used only for calculating *R*-factors(gt) *etc*. and is not relevant to the choice of reflections for refinement. *R*-factors based on *F*^2^ are statistically about twice as large as those based on *F*, and *R*- factors based on ALL data will be even larger.

### Fractional atomic coordinates and isotropic or equivalent isotropic displacement parameters (Å^2^)

**Table:**

	*x*	*y*	*z*	*U*_iso_*/*U*_eq_	
C1	−0.0351 (2)	0.2895 (2)	0.7281 (2)	0.0369 (4)	
H1B	−0.0197	0.2081	0.8148	0.044*	
C2	0.1044 (2)	0.34189 (18)	0.66995 (17)	0.0283 (3)	
C3	0.08495 (18)	0.46559 (17)	0.53574 (17)	0.0259 (3)	
C4	0.2268 (2)	0.5223 (2)	0.47013 (19)	0.0337 (4)	
H4A	0.3383	0.4773	0.5148	0.040*	
C5	0.2021 (2)	0.6413 (2)	0.3430 (2)	0.0406 (4)	
H5A	0.2965	0.6774	0.3026	0.049*	
C6	0.1682 (3)	0.0620 (3)	0.2747 (3)	0.0628 (7)	
H6A	0.2490	−0.0343	0.3287	0.094*	
H6B	0.1388	0.0527	0.1800	0.094*	
H6C	0.0643	0.0818	0.3284	0.094*	
C7	0.2506 (2)	0.1971 (2)	0.2543 (2)	0.0429 (4)	
H7A	0.2742	0.2097	0.3512	0.052*	
C8	0.1320 (3)	0.3506 (3)	0.1613 (3)	0.0607 (6)	
H8A	0.1903	0.4329	0.1461	0.091*	
H8B	0.0266	0.3784	0.2112	0.091*	
H8C	0.1040	0.3377	0.0677	0.091*	
C9	0.6308 (4)	0.2023 (4)	0.3419 (3)	0.0714 (8)	
H9A	0.5432	0.2048	0.4137	0.107*	
H9B	0.7008	0.2709	0.3505	0.107*	
H9C	0.7036	0.0964	0.3585	0.107*	
C10	0.5439 (3)	0.2570 (2)	0.1895 (2)	0.0437 (5)	
H10A	0.4767	0.3676	0.1709	0.052*	
C11	0.6760 (3)	0.2441 (4)	0.0729 (3)	0.0661 (7)	
H11A	0.7547	0.3062	0.0787	0.099*	
H11B	0.6168	0.2822	−0.0227	0.099*	
H11C	0.7404	0.1356	0.0886	0.099*	
N1	0.42071 (17)	0.15825 (17)	0.18060 (16)	0.0335 (3)	
H1C	0.3967	0.1694	0.0849	0.040*	
H1D	0.4761	0.0560	0.2216	0.040*	
O1	0.28340 (19)	0.14639 (18)	0.90458 (15)	0.0519 (4)	
O2	0.3674 (2)	0.38559 (19)	0.79367 (18)	0.0585 (4)	
O3	0.42442 (18)	0.17516 (17)	0.67405 (16)	0.0517 (4)	
S1	0.31154 (5)	0.25661 (5)	0.76799 (5)	0.0357 (2)	

### Atomic displacement parameters (Å^2^)

**Table:**

	*U*^11^	*U*^22^	*U*^33^	*U*^12^	*U*^13^	*U*^23^
C1	0.0390 (9)	0.0366 (9)	0.0327 (9)	−0.0133 (7)	0.0020 (7)	−0.0025 (7)
C2	0.0264 (7)	0.0285 (7)	0.0287 (8)	−0.0033 (6)	−0.0008 (6)	−0.0096 (6)
C3	0.0231 (7)	0.0279 (7)	0.0276 (7)	−0.0059 (6)	−0.0001 (6)	−0.0101 (6)
C4	0.0227 (7)	0.0399 (9)	0.0384 (9)	−0.0087 (6)	−0.0004 (6)	−0.0101 (7)
C5	0.0328 (9)	0.0468 (10)	0.0426 (10)	−0.0186 (8)	0.0049 (7)	−0.0054 (8)
C6	0.0394 (11)	0.0591 (14)	0.0805 (17)	−0.0143 (10)	0.0068 (11)	−0.0030 (12)
C7	0.0350 (9)	0.0497 (11)	0.0442 (10)	−0.0058 (8)	0.0055 (7)	−0.0187 (8)
C8	0.0441 (12)	0.0442 (11)	0.0883 (18)	0.0052 (9)	0.0023 (11)	−0.0255 (12)
C9	0.0689 (16)	0.118 (2)	0.0499 (13)	−0.0474 (16)	0.0010 (11)	−0.0383 (14)
C10	0.0414 (10)	0.0422 (10)	0.0495 (11)	−0.0152 (8)	−0.0024 (8)	−0.0114 (8)
C11	0.0519 (13)	0.105 (2)	0.0431 (12)	−0.0390 (13)	0.0018 (10)	−0.0054 (12)
N1	0.0291 (7)	0.0354 (7)	0.0345 (7)	−0.0038 (6)	−0.0022 (5)	−0.0115 (6)
O1	0.0527 (9)	0.0584 (9)	0.0318 (7)	−0.0073 (7)	−0.0081 (6)	0.0023 (6)
O2	0.0545 (9)	0.0595 (9)	0.0644 (10)	−0.0164 (7)	−0.0242 (7)	−0.0182 (8)
O3	0.0412 (8)	0.0515 (8)	0.0456 (8)	0.0119 (6)	0.0027 (6)	−0.0090 (6)
S1	0.0306 (3)	0.0390 (3)	0.0310 (3)	−0.00070 (17)	−0.00660 (16)	−0.00666 (18)

### Geometric parameters (Å, °)

**Table:**

C1—C2	1.366 (2)	C8—H8A	0.9600
C1—C5^i^	1.409 (3)	C8—H8B	0.9600
C1—H1B	0.9300	C8—H8C	0.9600
C2—C3	1.430 (2)	C9—C10	1.507 (3)
C2—S1	1.7844 (17)	C9—H9A	0.9600
C3—C4	1.422 (2)	C9—H9B	0.9600
C3—C3^i^	1.430 (3)	C9—H9C	0.9600
C4—C5	1.360 (3)	C10—C11	1.508 (3)
C4—H4A	0.9300	C10—N1	1.513 (2)
C5—C1^i^	1.409 (3)	C10—H10A	0.9800
C5—H5A	0.9300	C11—H11A	0.9600
C6—C7	1.508 (3)	C11—H11B	0.9600
C6—H6A	0.9600	C11—H11C	0.9600
C6—H6B	0.9600	N1—H1C	0.9000
C6—H6C	0.9600	N1—H1D	0.9000
C7—N1	1.508 (2)	O1—S1	1.4578 (15)
C7—C8	1.517 (3)	O2—S1	1.4401 (16)
C7—H7A	0.9800	O3—S1	1.4521 (15)
			
C2—C1—C5^i^	120.17 (16)	H8B—C8—H8C	109.5
C2—C1—H1B	119.9	C10—C9—H9A	109.5
C5^i^—C1—H1B	119.9	C10—C9—H9B	109.5
C1—C2—C3	121.06 (15)	H9A—C9—H9B	109.5
C1—C2—S1	118.57 (13)	C10—C9—H9C	109.5
C3—C2—S1	120.34 (12)	H9A—C9—H9C	109.5
C4—C3—C3^i^	118.72 (18)	H9B—C9—H9C	109.5
C4—C3—C2	123.01 (14)	C9—C10—C11	111.56 (19)
C3^i^—C3—C2	118.28 (17)	C9—C10—N1	109.39 (17)
C5—C4—C3	121.05 (15)	C11—C10—N1	109.33 (17)
C5—C4—H4A	119.5	C9—C10—H10A	108.8
C3—C4—H4A	119.5	C11—C10—H10A	108.8
C4—C5—C1^i^	120.72 (16)	N1—C10—H10A	108.8
C4—C5—H5A	119.6	C10—C11—H11A	109.5
C1^i^—C5—H5A	119.6	C10—C11—H11B	109.5
C7—C6—H6A	109.5	H11A—C11—H11B	109.5
C7—C6—H6B	109.5	C10—C11—H11C	109.5
H6A—C6—H6B	109.5	H11A—C11—H11C	109.5
C7—C6—H6C	109.5	H11B—C11—H11C	109.5
H6A—C6—H6C	109.5	C7—N1—C10	115.64 (14)
H6B—C6—H6C	109.5	C7—N1—H1C	108.4
N1—C7—C6	108.75 (16)	C10—N1—H1C	108.4
N1—C7—C8	109.60 (17)	C7—N1—H1D	108.4
C6—C7—C8	111.47 (19)	C10—N1—H1D	108.4
N1—C7—H7A	109.0	H1C—N1—H1D	107.4
C6—C7—H7A	109.0	O2—S1—O3	113.49 (10)
C8—C7—H7A	109.0	O2—S1—O1	112.42 (10)
C7—C8—H8A	109.5	O3—S1—O1	111.45 (9)
C7—C8—H8B	109.5	O2—S1—C2	106.31 (8)
H8A—C8—H8B	109.5	O3—S1—C2	106.05 (8)
C7—C8—H8C	109.5	O1—S1—C2	106.53 (8)
H8A—C8—H8C	109.5		

Symmetry codes: (i) −*x*, −*y*+1, −*z*+1.

### Hydrogen-bond geometry (Å, °)

**Table:**

*D*—H···*A*	*D*—H	H···*A*	*D*···*A*	*D*—H···*A*
N1—H1C···O1^ii^	0.90	2.03	2.887 (2)	159
N1—H1D···O3^iii^	0.90	2.02	2.916 (2)	174
C9—H9A···O3	0.96	2.58	3.480 (3)	156
C6—H6A···O3^iii^	0.96	2.58	3.351 (3)	138
C11—H11C···O1^iii^	0.96	2.61	3.439 (4)	144
C11—H11B···O2^ii^	0.96	2.47	3.382 (3)	158

Symmetry codes: (ii) *x*, *y*, *z*−1; (iii) −*x*+1, −*y*, −*z*+1.
